# ApplyPolygenicScore: An R package for applying polygenic risk score models

**DOI:** 10.1016/j.gimo.2025.103467

**Published:** 2025-10-24

**Authors:** Nicole Zeltser, Rachel M.A. Dang, Rupert Hugh-White, Daniel Knight, Jaron Arbet, Paul C. Boutros

**Affiliations:** 1 Department of Human Genetics, University of California, Los Angeles, CA; 2 Jonsson Comprehensive Cancer Center, University of California, Los Angeles, CA; 3 Institute for Precision Health, University of California, Los Angeles, CA; 4 Department of Urology, University of California, Los Angeles, CA

**Keywords:** Body mass index, Cancer, Polygenic risk model, Polygenic risk score

## Abstract

**Purpose:**

A polygenic score (PGS) predicts an individual’s genetic predisposition to a complex trait. A PGS is created by estimating the relative contributions of multiple common variants to the overall trait, creating a polygenic risk model (PGM). The PGM is then applied by combining its weights with the genotypes of a specific individual to estimate individual-specific genetic predisposition. Genome-wide association studies have served as the basis for thousands of PGMs, leading to many studies associating PGSs with a range of outcomes.

**Methods:**

To simplify, improve, and automate this task, we developed *ApplyPolygenicScore,* an open-source R package for applying standardized PGMs to new genetic data. We demonstrate its capabilities in a case study, applying a PGM for body mass index (BMI) in 1071 patients diagnosed with bladder, liver, and endometrial cancer.

**Results:**

*ApplyPolygenicScore* includes functions for input validation, allele matching, and PGS computation and visualization and is extensively documented. The computed PGS for BMI predicted BMI in patients with cancer, but its low accuracy indicates a larger role for nongenetic factors in BMI-influenced cancer outcomes.

**Conclusion:**

*ApplyPolygenicScore* encourages the wider research community to extend the findings of the statistical genetics niche, facilitating broader use of PGSs and subsequent novel discovery.

## Introduction

Thousands of genome-wide association studies (GWAS) have established that many phenotypes, including risk for most diseases, are influenced by genotypes at a large number of loci.^1^^,^^2^ One way of quantifying this association between genotypes at many alleles and a phenotype is a polygenic risk score (commonly abbreviated PRS or PGS). We conceptualize the PGS as a prediction of genetic risk in a single individual. GWASs initialize this process by estimating the effect of every analyzed variant on a trait in a population-scale cohort, most commonly through linear modeling. This model is typically adjusted before application, for example, by shrinkage of effect sizes to limit overfitting.^3^ In a form of feature selection, variants are retrained or removed based on independence (eg, with linkage-disequilibrium) and association to the phenotype (eg, *P* value or effect-size thresholding).^3^ Variants present in the population at any frequency may have a significant effect on a phenotype. However, highly penetrant rare variants are typically excluded from GWASs, and only lower effect-size common variants are incorporated into polygenic risk score models. The final model is applied to a new set of genotypes in an individual to make a prediction: the PGS. Because most GWASs are modeled linearly, individual risk variants are assumed to have an independent and additive influence on risk. Thus, the PGS is computed as the sum of the number of variant risk alleles carried by an individual, weighted by the modeled effect size of each individual allele. The polygenic risk model and its prediction are commonly referred to interchangeably with the term “PGS.” For clarity, we will refer to a set of variants and their estimated effect sizes as a polygenic risk model (PGM). We reserve the term PGS for the weighted sum resulting from the application of a PGM to an individual.

As GWAS have become more numerous and well powered, repositories such as the PGS Catalog^4^^,^^5^ have facilitated routine publication of PGMs in standardized formats. This growing availability of PGMs has facilitated investigations of polygenic risk beyond statistical genetics. Several clinical trials are now underway to determine clinical actionability of specific PGSs,^6^^,^^7^ and studies are beginning to characterize PGSs and their phenotypic correlates in biobank-scale cohorts.^8^, ^9^, ^10^ The implementation of a GWAS and training of a PGM is technical and computationally demanding, and many researchers are focused on the simpler process of applying existing PGMs to new genetic data. Facile application of an existing PGM requires software and workflows that are accessible to the computational novice yet robustly featured, including key quality-control diagnostics.

Software tools exists for the implementation of all parts of the PGS workflow,^5^^,^^11^^,^^12^ from model training to application, although most are designed primarily for the former. These tools, particularly the ubiquitous PLINK^11^ toolset, often require command-line expertise to implement. Their technical documentation can be difficult to parse for users interested in solely PGM application. For example, although the PGS Catalog’s pgsc_calc^5^ is designed specifically for PGM application, it is intended for command-line execution and requires technically demanding dependencies, such as Nextflow and Docker.^5^ Beyond computation of PGSs, a series of statistical analyses and visualizations are needed to facilitate interpretation. Custom scripts in the R statistical programming language are widely used for this purpose. This requires significant effort to import, format, and integrate data into R before this downstream analysis. A lack of standard visualization frameworks means that each analyst or group will use their own bespoke ways of assessing and quality-controlling the relationships between PGSs and phenotypes.

To fill these gaps, we created *ApplyPolygenicScore:* an easy-to-install, easy-to-understand and easy-to-use R package for the application of polygenic risk score models to genetic data. As an R package, *ApplyPolygenicScore* can be smoothly incorporated into the R environment-based analysis workflows favored by the genetics and biostatistics research community. Our tool imports genetic data in Variant Call Format (VCF) and PGS Catalog weight files, validates input data and parameters, performs allele matching, computes PGSs, and reports quality control metrics. With built-in plotting functionality, it integrates PGS and phenotype data into informative visualizations to facilitate explorative analysis and aid planning of subsequent statistical workflows. We demonstrate the utility of *ApplyPolygenicScore* by exploring the association of body mass index (BMI) PGSs with observed BMI in 1071 patients with cancer for 3 cancer types.^13^

## Materials and Methods

### Package implementation and recommended usage

**Figure 1:**
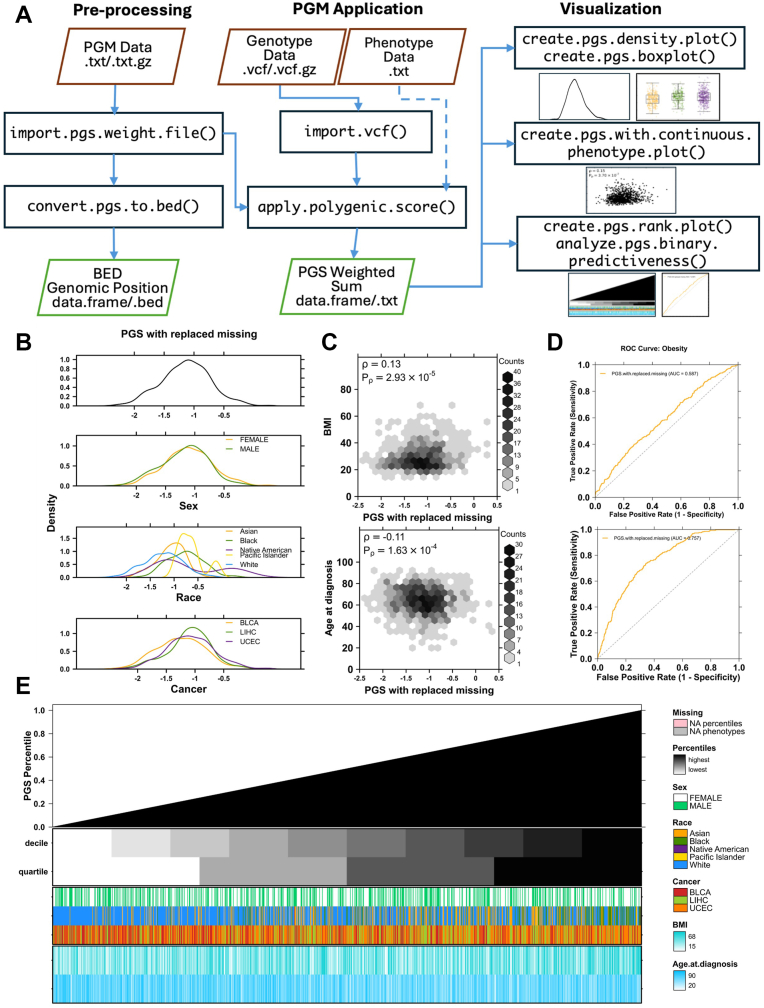
***ApplyPolygenicScore* functionality.** Application of *ApplyPolygenicScore* functions demonstrated in a case study of 1071 individuals from the TCGA database, diagnosed with bladder (BLCA), liver (LIHC), or uterine (UCEC) cancer. A. Recommended workflow when implementing functions provided by *ApplyPolygenicScore*. A set of preprocessing functions convert polygenic risk score model (PGM) weight files into BED-formatted genomic coordinate files for suggested use in filtering VCF genotype data to desired coordinates. PGM application functions facilitate genetic data importation and weighted sum computation. Visualization functions provide summary information on computed PGSs and phenotype data. Solid arrows indicate required inputs and dotted arrows indicate optional inputs. B. BMI PGS densities, cohort-wide and by categorical phenotypes, computed in the case study cohort and automatically plotted by the create.pgs.density.plot() function. C. Correlations of PGSs from (B) with continuous phenotypes automatically plotted by the create.pgs.with.continuous.phenotype.plot() function. D. Receiver-operator curves plotted by the analyze.pgs.binary.predictiveness() function depicting the performance of the PGSs from (B) to predict obesity status as a sole predictor (top) and with covariates age at diagnosis, sex, and the first 10 principal components of genetic ancestry (bottom). Positive obesity status is defined as BMI ≥ 30. E. From top to bottom: percentile rank of PGSs from (B) for each individual in ascending order, decile and quartile covariate bars, categorical phenotype covariate bars, and continuous phenotype heatmaps.

The *ApplyPolygenicScore* package is written in the R language and implemented in the R software environment. It is available for installation through CRAN and GitHub. It comprises ∼8000 lines of R code, with over 700 unit tests and systematic input and output parameter validation. *ApplyPolygenicScore* provides a suite of functions designed to facilitate a simple and reproducible workflow for the application of an existing PGM to genetic data in VCF. This functionality is equivalent to the --score flag in the standard PLINK^11^ toolset but within the convenience of the R environment and with more robust handling of genetic data edge cases (Supplemental Figures 1-2, Supplemental Tables 1-2). *ApplyPolygenicScore* supports 3 categories of workflows: input preprocessing, PGM application and visualization (Figure 1A).

### Preprocessing

Genetic data files, whether from sequencing, genotyping, or imputation, are often very large and require significant computer memory (ie, RAM). To preempt the possibility of insufficient memory in the R environment to process genome-scale genetic inputs, *ApplyPolygenicScore* takes advantage of the fact that the number of informative variants encompassed by most PGMs is orders of magnitude smaller than the total number of polymorphic regions in the genome (Supplemental Figure 1A). Our package provides functionality to produce BED-formatted files of genomic coordinates for PGM component variants, which can easily be used to filter genetic data to a much more manageable size before importation into R.

### Application of PGS to genetic data

The application of an existing polygenic score model to new genetic data begins with the matching of genotype data to weights for each PGM variant. This is followed by computation of the dosage for each individual at each variant site, relative to each variant’s effect allele. When performing dosage computation, *ApplyPolygenicScore* inherits the most common GWAS assumptions: (1) that the effect of each variant is independent of all others and (2) that the effects of different variants are linearly additive. Dosage at a variant is equal to the number of effect alleles at that variant. Each dosage is multiplied by the corresponding variant’s weight, then, according to the additive model of polygenic risk, all weighted dosages for an individual are summed together to produce the final PGS. Equation 1 depicts this framework for individual *i* and variant *m* of *M* total variants.

$$1.\;PGS_{i}=\sum_{m=1}^{M}\beta_{m}\times dosage_{im}$$

*ApplyPolygenicScore* simplifies this process by standardizing the format of input genotype and PGM weight data, providing functions for performing the matching, dosage calculation, and weighting and summation steps, accounting for multiallelic sites, and providing various options for handling strand flip verification and missing genotype data. The resulting computed scores accurately replicate equivalent options implemented by similar tools, such as PLINK^11^ (a command-line library for genetic data analysis), pgsc_calc^5^ (a command-line companion tool to the PGS Catalog) and bigsnpr^14^ (an R library for genetic data analysis; Supplemental Figure 1B-C, Supplemental Tables 1-2). Notably, *ApplyPolygenicScore* handles multiallelic edge cases more robustly than these tools because it does not rely on PLINK-formatted genetic data file types (Supplemental Figure 1B-C, Supplemental Table 1). PLINK duplicates records for multiallelic sites, causing complications when matching genotype information to PGM weight information (Supplemental Figure 2). Both pgsc_calc and bigsnpr inherit these problems from their input format (pgsc_calc internally converts VCF to PLINK). This can result in incorrect dosage calculations, visible as a slight discordance between certain samples compared in Supplemental Figure 1B-C. Dosage calculation in *ApplyPolygenicScore* is performed in an allele-aware manner, allowing intuitive handling of effect-size flips. This feature is not easily automated by the bigsnpr library, resulting in a reversed effect-size direction (Supplemental Figure 1C).

VCF file importation is supported *via* vcfR,^15^ with a utility function import.vcf() for standardizing and verifying import parameters. The memory and runtime required by the main apply.polygenic.score() function scales linearly with the product of the number of samples in the cohort (n) and the number of variants in the PGM (m), making our implementation O(nm) in both space and time (Supplemental Figure 1D). Clinical research cohorts typically contain tens to tens of thousands of samples.^8^^,^^16^, ^17^, ^18^ A typical input cohort of 1000 samples and an input PGM with 1000 variants (creating a matrix of size 10^6^) requires under 1GB of RAM and under 10 seconds to complete, comparable to existing tools (Supplemental Figure 1D). An input matrix of size 10^9^ (eg, 1000 samples with 1 million variants) requires approximately 130 GB and under 2 hours to complete.

Because of their varying ploidy and the presence of pseudo autosomal regions (PARs), the sex chromosomes represent a unique challenge for both variant detection^19^ and PGM training and application. These complexities have resulted in the frequent exclusion of X and Y variants from GWAS and PGMs, and a lack of standards on how sex-chromosome dosage should be interpreted.^20^ Genotypes in VCF across the X and Y chromosomes can be formatted as haploid or diploid. Male samples can often include diploid calls in haploid regions, depending on variant caller settings (often assuming diploid ploidy in all chromosomes in mixed-sex cohorts) and postprocessing decisions. *ApplyPolygenicScore* interprets sex-chromosome genotypes as given in the VCF input (applying a dosage of 1 to hemizygous effect allele calls), thus relying on the user to apply their desired formatting. Providing additional options for PAR- and sex-sensitive dosage interpretation is a key area of future development in *ApplyPolygenicScore*.

*ApplyPolygenicScore* facilitates an easy transition to downstream analysis. Individual PGS percentiles (relative to the full cohort) and missing variant counts are computed and reported alongside PGSs. If phenotype data are provided, a statistical association is automatically computed between the PGSs and any user-defined phenotype. This is particularly useful for evaluating the accuracy of a PGS to predict the phenotype it is trained on. Phenotype data are automatically aggregated with PGSs in the final output, ready for use in further analyses and visualizations.

### Visualization

The package provides 5 plotting functions that produce convenient visualizations from the output of apply.polygenic.score() (Figure 1B-E). These are create.pgs.density.plot() and create.pgs.boxplot() (plotting PGS distributions as density curves or boxplots, optionally by categorical phenotype), create.pgs.with.continuous.phenotype.plot() (plotting PGS values against continuous phenotypes as a scatter plot or hexbin plot), create.pgs.rank.plot() (plotting ranked PGS percentiles, optionally with missingness counts and covariate bars indicating categorical or continuous phenotypes) and analyze.pgs.binary.predictiveness() (performing a logistic regression against a binary or dichotomized continuous phenotype and returning area under the curve statistics along with a plotted receiver-operator curve). These plots provide at-a-glance assessments of scoring quality and insights into genotype-phenotype relationships. Each plotting function can be used with any data set in R, providing compatibility with the output of any other tool. All plotting functionality is built on top of the BoutrosLab.plotting.general^21^ package (v7.1.0).

### Benchmarking analysis

#### Benchmarked tools

Three tools were chosen for comparison with *ApplyPolygenicScore* (v4.0.0): pgsc_calc^5^ (v2.1.0; a command-line tool for PGM application that accompanies the PGS Catalog), PLINK2^11^ (v2.0.0-a.6.5LM; an extensive command-line library for various genetic data analysis tasks), and bigsnpr^14^ (v1.12.18; an R package with an extensive library of genetic data analysis functions).

#### PGS correlation analysis

An identical polygenic risk score model was applied to an identical set of genotypes by all tools using equivalent parameters. Both inputs were the same as those evaluated in the case study and are described in detail (see Materials and Methods, Case Study Data and Analysis section). The PLINK2 toolset was used to convert files in VCF to the PLINK2 format required by PLINK2 and the PLINK1.9 format required by bigsnpr. Neither PLINK2 nor bigsnpr provide automated parsing of a PGS Catalog formatted weight file; therefore, this input was reformatted accordingly in R.

#### Memory and runtime benchmarking

The publicly available 1000 Genomes (1KG) data set^22^ (GRCh38, phase 3) was used to create 5 sets of genotype input files spanning 5 input file sizes (25 total). A total of 1000 1KG samples were randomly selected for analysis. The 1KG variant set was filtered to keep only biallelic SNPs. The final benchmarking set was created by randomly selecting variants 5 times for each of the 5 input sizes: 10^2^, 10^3^, 10^4^, 10^5^, and 10^6^. For each genotype file, a mock PGM weight file was created with 1 variant for each genotype coordinate. Because each input contained 10^3^ samples, the benchmarked input sample by single-nucleotide polymorphism (SNP) matrix sizes were 10^5^, 10^6^, 10^7^, 10^8^, and 10^9^. PLINK2 was used to convert genotype inputs into the file formats required by various tools (VCF, PLINK1.9, PLINK2; Supplemental Table 1). The tool pgsc_calc was implemented with a VCF input.

The workflow manager Nextflow’s trace functionality was used to produce comparable measurements of memory requirements and runtime. Equivalent PGM application scripts using *ApplyPolygenicScore*, PLINK2, and bigsnpr were executed within a Nextflow (v23.04.2) workflow, producing a trace file with realtime and peak resident set size statistics. The tool pgsc_calc is itself a Nextflow (v24.10.4) pipeline and was executed according to tool instructions. The reported pgsc_calc trace file contains statistics from 7 consecutive tasks. The maximum reported peak resident set size value and the sum of all reported realtime values were used to represent pgsc_calc performance with a single statistic.

### Case study data and analysis

#### Cohort

The BMI analysis cohort was established by filtering The Cancer Genome Atlas (TCGA) pan-cancer clinical data for individuals with complete (nonmissing) height and weight values, as well as available genotype array data. After this filtering, the 3 most abundant remaining cancer types were selected for analysis: bladder urothelial carcinoma (BLCA) (*n* = 320), liver hepatocellular carcinoma (LIHC) (*n* = 308), and uterine corpus endometrial carcinoma (UCEC) (*n* = 461). The following exclusions were applied to create the final cohort: individuals with a cancer diagnosis before 18 years of age (suggestive of pediatric cancers), unusually short height (height ≤ 70 cm), a history of neoadjuvant treatment or a cancer belonging to a rare distinct histological subtype (Fibrolamellar Carcinoma). After exclusions, the cohort included 311 BLCA, 303 LIHC, and 457 UCEC patients, for a total of 1071.

#### BMI

BMI was calculated from weight and height data according to the formula: weight (kg)/(height (m)).^2^

#### Genotype data processing

TCGA germline data from array genotyping, reported against the GRCh37 human reference genome, had been previously converted to VCF, quality controlled, and stranded to the 1KG reference panel.^23^ Starting with these data, we performed imputation using the 1KG phase 3 reference panel in GRCh37 through the Michigan Imputation Server.^22^^,^^24^ Resulting per-chromosome VCF files were concatenated using bcftools (v1.15.1)^25^ and filtered for individuals belonging to the selected BMI analysis cohort. The PGM for BMI was selected for being relatively current (published in 2023), having been trained in a large population (>400,000; UK Biobank^26^) and encompassing a large number of variants (1127). The harmonized weight file for GRCh37 was downloaded from the PGS catalog^4^^,^^5^ under the ID: PGS004609. The *ApplyPolygenicScore* function convert.pgs.to.bed() was used to create a BED-formatted genomic coordinate file for all variants in the PGM. This file was used to filter the cohort VCF file for PGM variants. Finally, all multiallelic sites were merged using bcftools to comply with *ApplyPolygenicScore’s* genotype data formatting requirements.

#### Application of the PGM

The BMI PGM was applied using the *ApplyPolygenicScore* R package (v3.0.1). VCF data and PGM data were imported into R (v4.2.0) using the import.vcf() and import.pgs.weight.data() functions, respectively. Allele matching and strand matching between the VCF and PGM data was verified by first merging the data using combine.vcf.with.pgs() then by checking allele concordance via assess.pgs.vcf.allele.match(). PGSs were then computed in each individual using the apply.polygenic.score() function with the mean.dosage missing genotype method and strand flip correction disabled. The “mean.dosage” method replaces missing genotypes with a mean dosage computed from nonmissing genotypes and returns a data column named “PGS.with.replaced.missing.” Because no genotypes remained missing after imputation, no mean dosage replacements needed to be executed by the method.

#### Statistical analysis

All statistical analyses were performed in R (v4.2.0). Principal components (PCs) of genetic ancestry were computed by first merging cohort genotypes with the 1KG reference cohort (phase 3 primary release, genome build GRCh37, founders only) using bcftools. Merged genotypes were converted to PLINK2 format and filtered for a maximum missingness of 0.05 and a minimum allele frequency of 0.01 and then pruned for variants in high linkage disequilibrium. PCs in the merged cohort were computed using the PLINK2 --pca flag.

The analyze.pgs.binary.predictiveness() function in *ApplyPolygenicScore* was used to generate receiver-operator curves (ROCs) and compute area under the receiver-operator curve (AUC) statistics. This function utilizes R’s glm() function to fit a logistic regression model and the roc() function from the pROC package to compute ROC components. One ROC analysis was performed with the PGS as the sole predictor and a second ROC analysis was performed with sex, age at diagnosis, and the first 10 PCs of ancestry as covariates to the logistic regression. The dependent phenotype was obesity status. Positive obesity status was defined as BMI ≥ 30.

A multivariable linear regression analysis predicting observed BMI from a PGS for BMI was performed within BLCA, LIHC, and UCEC cohorts. This regression was adjusted for age at diagnosis, race, and the first 10 PCs of ancestry. Race was defined as a binary variable with White as the reference level and non-White as the comparison level. A second multivariable linear regression was performed in BLCA and LIHC cohorts, adjusting for sex in addition to all previous covariates. Sex was defined with male as the reference level and female as the comparison level.

Data in scatter plots depicting smoothed lines were smoothed using the locally estimated scatter plot smoothing (loess) method. Delta (change) in predicted BMI compared with observed BMI was computed as residuals from fitting a simple linear model between computed PGSs and observed BMI. Differences in BMI, PGSs, or BMI delta between more than 2 groups were computed using the Kruskal-Wallis test and between 2 groups using the Wilcoxon Rank-Sum test. All tests were 2-sided with an alpha level of 0.05 indicating statistical significance. The Bonferroni method for multiple testing correction was applied to all sets of pairwise tests between more than 2 groups.

#### Visualization

All visualizations were created using the *ApplyPolygenicScore* package (v3.1.0) or the *BoutrosLab.plotting.general* (v7.1.0) package in R (v4.2.0). In various boxplots, asterisks were plotted to represent statistical significance between 2 groups. One asterisk represents *P* < .05, 2 represent *P* < .01, and 3 represent *P* < .001.

## Results

To demonstrate the capabilities of *ApplyPolygenicScore,* we evaluated a polygenic risk score model for BMI^27^ in a cancer cohort. BMI has been explored as a potential risk factor in several cancer types, but sparse recording of BMI in clinical cohorts can make investigations challenging. A PGS for BMI, trained in a healthy population, represents a healthy person’s genetic predisposition to a specific BMI. In clinical cohorts in which genetic data are available, this PGS may be a useful alternative for measured BMI and may provide insights into genetic drivers of BMI-related cancer risk.

After exclusions, we analyzed genetic and clinical data from 1071 individuals (Table 1) diagnosed with BLCA (*n* = 311), LIHC (*n* = 303), and UCEC (*n* = 457).^28^, ^29^, ^30^ A 1127-variant PGM for BMI, trained in the UK Biobank, was selected for analysis.^26^ Some variants (141; 12.5%) were directly measured; the remainder were imputed (see Materials and Methods).

**Table 1:** Characteristics of the TCGA cohort used for evaluation of a polygenic risk score (PGS) model for body mass index (BMI)

	Bladder Cancer (BLCA) (*N* = 311)	Liver Cancer (LIHC) (*N* = 303)	Uterine Cancer (UCEC) (*N* = 457)	Total (*N* = 1071)
BMI				
Median (Q1-Q3)	25.7 (23.0-29.3)	24.4 (21.7-28.5)	32.4 (26.6-39.1)	27.3 (23.6-33.7)
Age at diagnosis				
Median (Q1-Q3)	68.0 (60.0-76.0)	61.0 (52.0-68.0)	63.0 (57.0-71.0)	64.0 (56.0-72.0)
Sex				
Male	228 (73.3%)	208 (68.6%)	0 (0%)	436 (40.7%)
Female	83 (26.7%)	95 (31.4%)	457 (100%)	635 (59.3%)
Race				
White	242 (77.8%)	131 (43.2%)	317 (69.4%)	690 (64.4%)
Asian	40 (12.9%)	150 (49.5%)	19 (4.2%)	209 (19.5%)
Black	14 (4.5%)	14 (4.6%)	90 (19.7%)	118 (11.0%)
Pacific Islander	0 (0%)	0 (0%)	6 (1.3%)	6 (0.6%)
Native American	0 (0%)	1 (0.3%)	2 (0.4%)	3 (0.3%)
Missing	15 (4.8%)	7 (2.3%)	23 (5.0%)	45 (4.2%)

### PGS application with *ApplyPolygenicScore*

First, we verified variant- and allele matching quality between the PGM and VCF data using the combine.vcf.with.pgs() and assess.pgs.vcf.allele.match() functions. No PGM variant coordinates were missing from the VCF data (Supplemental Tables 3-4). A majority of variants (947; 84%) were identified as having effect sizes reported relative to the reference allele in the VCF data, as opposed to the alternative allele (labeled “effect_switch”; Supplemental Table 3). This case is handled by *ApplyPolygenicScore*’s dosage calculation functionality because weights are assigned to alleles by their alphabetic base, agnostic of whether they are labeled reference (REF) or alternative (ALT) in a VCF. The remaining 180 variants (16%) were identified as palindromic. Because it is impossible to distinguish a strand flip from an effect-size flip in a palindromic variant based solely on allele comparison, these variants were flagged as ambiguous strand flip cases (labeled “ambiguous_flip”; Supplemental Table 3). *ApplyPolygenicScore* provides the option to automatically detect such variants and discard them. Because no strand flips or allele mismatches were detected in any nonpalindromic variant, we determined that these cases were unlikely to represent true strand flips and proceeded to PGM application. This optional check can be automatically performed by the apply.polygenic.score() function with the max.strand.flips parameter.

Next, we used the apply.polygenic.score() function to apply the BMI PGM to the analysis cohort, which also automatically performed a simple linear regression analysis of the computed PGSs against observed BMI (Supplemental Tables 4-5). The PGSs were predictive of BMI in the full cohort but displayed low accuracy (*P* < .001, R^2^ = 0.03, univariable linear regression; Supplemental Table 5). Figures 1B-E show plots generated by visualization functions included in *ApplyPolygenicScore*. The PGS distribution appeared to vary substantially by self-reported race (Figure 1B). The density curve of PGSs among Black participants was shifted to the right of the curves corresponding to Asian and White participants (Figure 1B). Such shifts are a documented consequence of applying a PGM to individuals who are genetically dissimilar to the population in which the PGM was trained.^31^ The PGM for BMI used here was originally trained exclusively in individuals with inferred European ancestry,^26^ making it less accurate for participants who are less genetically similar to that population and introducing systematic ancestry bias. Because race often correlates with genetic ancestry, we inferred that our cancer cohort likely spanned a spectrum of ancestral genetic variation. To account for genetic ancestry-driven bias in the PGM, we computed a continuous measure of ancestry in the form of genetic PCs for use in downstream multivariable analyses (see Materials and Methods). The PGSs had a small but significant positive correlation with BMI, and a negative correlation with age at cancer diagnosis (Figure 1C). No obvious patterns between PGS percentile rank and phenotypes were revealed from visual inspection of Figure 1E. Figure 1D shows the results of an automated case-control study style analysis performed by the function analyze.binary.predictiveness(). We used the function with a BMI threshold of 30 to automatically convert the continuous BMI phenotype into a binary positive (BMI ≥ 30) and negative (BMI < 30) obesity status phenotype. PGS as the only predictor produced the receiver-operator curve in the upper panel of Figure 1D, with an AUC of 0.59. When the underlying logistic regression was adjusted for covariates (age at diagnosis, sex, and 10 PCs of genetic ancestry), the AUC of the model increased to 0.76. Taken together, the results of this fully automated exploratory analysis confirmed that a PGM for BMI remained predictive of BMI and obesity status in a cohort of patients with cancer. Additionally, race was corroborated as a known potential confounder to polygenic risk analyses and a potential association was indicated between genetic BMI predisposition and earlier age of cancer diagnosis.

### Accuracy of BMI PGSs in cancer cohorts

**Figure 2:**
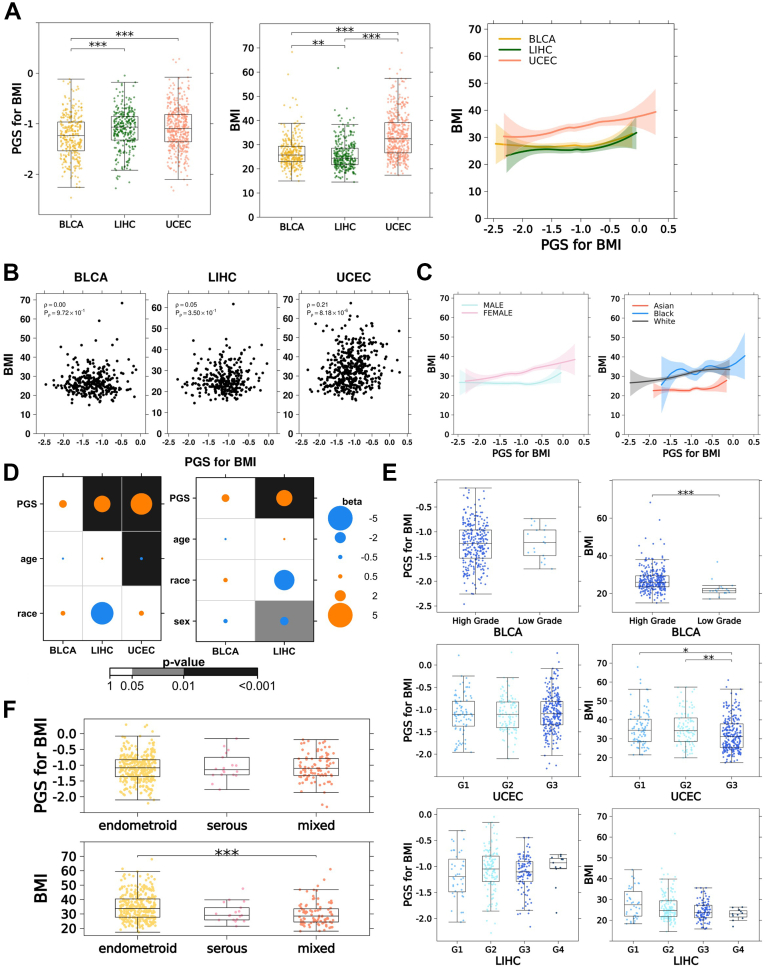
**BMI PGS in cancer cohorts.** Analysis of BMI PGS computed by *ApplyPolygenicScore* in a case study of 1071 individuals from the TCGA database, diagnosed with bladder (BLCA), liver (LIHC), or uterine (UCEC) cancer. A. Per-cancer distributions of computed PGS for BMI (left) and recorded BMI (middle) and loess-smoothed curves between PGS for BMI and BMI (right). Shaded regions indicate 95% confidence intervals. B. Per-cancer correlation between computed PGS for BMI and recorded BMI. C. Loess-smoothed curves between PGS for BMI and BMI by sex (left) and race (right). Shaded regions indicate 95% confidence intervals. D. Results of linear regression predicting observed BMI using computed PGS for BMI, adjusted by race, age at cancer diagnosis, and the first 10 principal components of ancestry (left) and additionally sex (right). E. Computed PGS for BMI and observed BMI in relation to cancer grade, by cancer type. F. Computed PGS for BMI and observed BMI in relation to histological subtypes of UCEC. In all boxplot panels, asterisks indicate statistically significant differences between indicated groups from pairwise Wilcoxon testing. *P* values are adjusted for multiple testing within each set of groups using the Bonferroni method. One, 2, and 3 asterisks indicate adjusted *P* value below .05, .01, and .001, respectively.

To investigate whether BMI PGS accuracy varies between patients with different cancers, we used the output of apply.polygenic.score(). Both PGSs for BMI and observed BMI differed significantly between at least 2 sets of cancer types (Figure 2A). The pairwise differences between LIHC and the other 2 cancer types were not concordant between PGS and BMI measurements. Every group significantly differed from the others in observed BMI (Bonferroni-adjusted *P*< .01, Wilcoxon tests), with LIHC having the lowest median BMI (Figure 2A). Among PGS distributions, LIHC had the highest median, and the LIHC distribution did not differ significantly from UCEC (Bonferroni-adjusted *P* = 1, Wilcoxon test). The significant correlation between the PGSs and BMI observed in the aggregated cohort was not replicated in BLCA and LIHC, with only UCEC patients showing a significant positive correlation (Figure 2A-B). Similarly, the BMI distributions of patients in the lowest and highest 5% of cohort-wide genetic risk only significantly differed in UCEC patients (*P* = .03, Wilcoxon test; Supplemental Figure 3A). The correlation between PGSs for BMI and observed BMI differed by sex and race (Figure 2C). The stronger correlation observed in women is consistent with the relationship observed in the female-exclusive UCEC cancer type. For an additional representation of PGS accuracy, we computed BMI delta for each patient: the residual between the observed and PGS-predicted BMI from fitting a simple linear model. BMI delta distributions were similar to those of BMI, indicating that variation in BMI by cancer type was not well captured by the PGS. No correlation was detected between age at diagnosis and BMI delta, indicating patient age likely did not affect PGS accuracy (Supplemental Figure 3B).

To assess the association of BMI PGS to observed BMI in the context of epidemiological variables, we performed multivariable linear regressions in each cancer type, with race (non-White vs White), age at diagnosis, and the first 10 principal components of genetic ancestry as covariates in all 3 cancer types and sex as an additional covariate in BLCA and LIHC. Model performance varied by cancer type. The sex-adjusted LIHC model was the most accurate at predicting BMI, with the highest R^2^ of 0.32. Predictions were least accurate in the UCEC cohort with an R^2^ of 0.15. Both BLCA models achieved an R^2^ of 0.17. The computed PGS was a significant positive predictor in LIHC and UCEC, but not BLCA (Figure 2D). Among all significant coefficients, PGS in the UCEC cohort showed the highest effect size (Figure 2D). Age at diagnosis was significantly associated with lower BMI in UCEC. Female sex was a significant predictor of lower BMI only in LIHC. Non-White race vs White race was not associated with BMI, corroborating that the race-related distribution patterns seen in Figure 1B were most likely caused by genetic ancestry bias in the PGM. These results indicate that although some of the variation in BMI in a patient with cancer can be attributed to genetic predisposition, many nongenetic variables contribute as well.

### BMI PGSs and cancer clinical features

Next, we explored how BMI PGSs were associated with clinical features of cancer. Significant differences in BMI by cancer grade were detected in BLCA and UCEC, none of which were replicated in PGS distributions (Figure 2E). Once again, BMI delta distributions were highly similar to BMI distributions (Supplemental Figure 3C). Among T-category (only reported in LIHC and BLCA) and N-category (only reported in BLCA) classifications, no differences were detected in PGSs, and LIHC was the only cancer type for which BMI varied by T-category (Supplemental Figure 3D-E). Uterine cancers arise as distinct histologic subtypes with different clinical and molecular characteristics. BMI differed significantly between UCEC endometroid and mixed subtypes, and this trend was not reflected in BMI PGSs (Figure 2F). The relationship between predicted and actual BMI was more consistently linear and positive in the endometroid subtype (Supplemental Figure 3F).

## Discussion

*ApplyPolygenicScore* is designed to make the specific task of applying an existing polygenic risk score model to genetic data more accessible to the wider research community. The acquisition of genetic data is becoming increasingly more affordable, and yet more is being shared in public and research-controlled data sets. For example, the PGS Catalog reported 5053 published PGSs in its February 4, 2025 release.^4^^,^^5^ As PGS models grow in both number and generalizability,^32^^,^^33^ it is becoming increasingly common to incorporate them into a wide range of studies. The association of PGSs with traits is the subject of a growing body of research, and *ApplyPolygenicScore* facilitates the cohesive implementation of all aspects of the required analysis workflow entirely in the statistical programming language R. In keeping with the expectation of multidisciplinary use cases, *ApplyPolygenicScore* documentation is written with the bioinformatics, genetics, and statistical layman in mind and includes vignettes that guide the user through basic analyses with real and simulated data.

In a case study of PGSs for BMI in patients with cancer, we demonstrated the utility of *ApplyPolygenicScore* as a component of a PGS-focused translational analysis workflow. Our tool successfully applied a 1127-variant PGM to 1,071 samples from a clinical cohort and generated informative visualizations. Our analysis revealed that a PGS for BMI can predict BMI in a cancer cohort, although with lower accuracy than in a healthy population.^26^ These findings imply that BMI measured shortly after cancer diagnosis is not equivalent to BMI in healthy individuals. This complicates the retrospective study of BMI in cancer cohorts because prediagnosis height and weight records are often unavailable. Prospective cancer studies that deliberately gather BMI data before diagnosis would make a valuable resource for the study of BMI-mediated cancer risk. Unlike the fluctuating BMI, a PGS for BMI represents a fixed measurement of inherited predisposition. The PGS for BMI generally failed to reproduce trends with clinical and demographic variables associated with observed BMI. Our results indicate that the genetic component of BMI encompassed by the tested PGM likely does not represent a large contribution of risk for more adverse cancer outcomes.

Although *ApplyPolygenicScore* is easily incorporated into R-based workflows, this brings limitations. R is not well equipped to handle very large data sets. Because all input data must be loaded into RAM, users will be limited by available memory. Because apply.polygenic.score memory requirements scale linearly with both sample size (*n*) and PGM component variant count (m), users attempting to apply very large PGMs may need to batch analyses. apply.polygenic.score runtime also scales linearly with the (nm) matrix. Inputs of size 10^8^ and below complete within a practical range of seconds to minutes. Larger inputs scale into hours, and users may also wish to batch analyses to save time. Approximately a quarter of the models in the PGS Catalog exceed 1 million variants. However, most genome-scale PGMs incorporate a large number of nonsignificantly associated variants, and the heritability of most traits is likely encompassed by many fewer variants.^34^ As GWAS increase in power, genome-scale PGMs will give way to smaller, more precise, and more accessible models. *ApplyPolygenicScore* is not designed to handle truly large-scale studies, and researchers in such cases can refer to an existing selection of highly optimized tools. For researchers investigating genetic risk in clinical studies, the typically smaller scale and precise clinical goal of such studies makes *ApplyPolygenicScore* a convenient and practical resource.

Another memory-linked limitation is the difficulty of implementing population-scale reference panel-based calibrations or biobank-scale adjustments. Such methods have been shown to increase PGS accuracy,^5^^,^^31^^,^^35^ but most involve adjustments to PGM weights during model training or adjustments to the PGS after it has been computed. The task performed by *ApplyPolygenicScore* remains separate and modular to these enhancements, and such methods can easily be incorporated before or after *ApplyPolygenicScore* is used. As methods become more efficient and user friendly, the potential exists for incorporating post-PGS-computation implementations into *ApplyPolygenicScore*.

The seemingly simple task of aggregating genetic risk in a PGS is made much more complex by the nature of biological systems. The method implemented to apply PGMs in *ApplyPolygenicScore* is currently limited to the ubiquitous additive model of polygenic risk. Alternative methods, accounting for dominance and epistatic effects do exist and show marginally improved prediction for some phenotypes.^36^^,^^37^ Should the application of these models become standardized in resources such as the PGS Catalog, *ApplyPolygenicScore* can be trivially updated with additional scoring methods. The additive model requires an accurate allele count, but the number of effect alleles in an individual’s genome can depend on structural features such as copy-number variation of DNA segments containing the effect allele. Because structural variation is not typically called or reported alongside SNPs or short indels in a VCF file, *ApplyPolygenicScore* does not currently include functionality to account for its effect on allele copy number. The GWASs upon which PGMs are trained also rarely adjust variant effect sizes for copy number; therefore, a standard for how this information would be integrated does not yet exist. As our understanding of genetic risk improves, there will be opportunities to modify *ApplyPolygenicScore* for more accurate PGM application.

### Conclusion

The *ApplyPolygenicScore* package provides an accessible toolkit for the application of polygenic risk score models to genetic data. This software can be used by the broad biological research community to advance understanding of genetic risk, in medical applications and beyond.

## Data Availability

*ApplyPolygenicScore* and associated documentation is available on GitHub (https://github.com/uclahs-cds/package-ApplyPolygenicScore/) and CRAN (https://cran.r-project.org/package=ApplyPolygenicScore).

Previously processed^23^ TCGA germline genotype VCF files stranded to the 1000 Genomes reference panel were downloaded from the Genomic Data Commons (GDC) portal on April 12, 2024 using the manifest provided by the original authors on the GDC-hosted web page pertaining to the CCG-AIM-2020 study (https://gdc.cancer.gov/about-data/publications/CCG-AIM-2020). Patient data identifiers were mapped to corresponding genetic data using the file Map_TCGAPatientID_BirdseedFileID.txt, which is available for open-access download on the same page. TCGA clinical data, including height and weight for BMI calculation, were downloaded on April 24, 2024 via open access from the GDC-hosted web page pertaining to the PanCanAtlas publications (https://gdc.cancer.gov/about-data/publications/pancanatlas) as part of the clinical_PANCAN_patient_with_followup.tsv file. PGS weight data were downloaded from the PGS catalog using PGS ID PGS004609. This score was released and harmonized in the PGS Catalog on February 20, 2024.

## Conflict of Interest

Paul C. Boutros is on the Scientific Advisory Boards of BioSymetrics Inc and Intersect Diagnostics Inc and was formerly on that of Sage Bionetworks. All other authors declare no conflicts of interest.
