# Supplementary material for: ApplyPolygenicScore: An R package for applying polygenic risk score models
Source: Genet Med Open. 2025 Oct 24;3:103467. doi: 10.1016/j.gimo.2025.103467 (PMC12755996; doi:10.1016/j.gimo.2025.103467)
Supplement: Supplementary Material [file mmc1.docx]

Supplementary Materials for ApplyPolygenicScore: An R package for applying polygenic risk score models

Table of Contents

[Supplementary Tables 2](#_Toc210997982)

[Supplementary Table 1. *ApplyPolygenicScore* feature comparison 2](#_Toc210997983)

[Supplementary Table 2. Equivalent PGM application workflows among benchmarked tools. 3](#_Toc210997984)

[Supplementary Figures 4](#_Toc210997985)

[Supplementary Figure 1. ApplyPolygenicScore benchmarking 4](#_Toc210997986)

[Supplementary Figure 2. Real data example of multiallelic site handling by ApplyPolygenicScore and PLINK. 6](#_Toc210997987)

[Supplementary Figure 3. BMI PGS and TCGA clinical features 8](#_Toc210997988)

# Supplementary Tables

## Supplementary Table 1. *ApplyPolygenicScore* feature comparison

Comparison of software characteristics and polygenic risk score model application features in *ApplyPolygenicScore* and similar tools.

|  | **ApplyPolygenic**  **Score** | **pgsc_calc** | **PLINK1.9/2** | **bigsnpr** |
| --- | --- | --- | --- | --- |
| **Software Features** | | | | |
| **Primary dependencies** | R | Nextflow,  environment manager *e.g.* Docker | PLINK1.9/2 | R |
| **Genetic input format** | VCF | VCF, PLINK1.9/2 | PLINK1.9/2 | PLINK1.9, BGEN |
| **Tool purpose** | PGM application | PGM application | General genetic data analysis | General genetic data analysis |
| **Built-in PGM Application Features** | | | | |
| **PGS Catalog compatibility** | ✓ | ✓ | ✗ | ✗ |
| **Input validation** | ✓ | ✓ | ✗ | ✗ |
| **Coordinate matching** | ✓ | ✓ | ✗ | ✓ |
| **Variant ID matching** | ✓ | ✗ | ✓ | ✗ |
| **Strand flip handling** | ✓ | ✓ | ✗*^a^* | ✓ |
| **Unmatched variant reporting** | ✓ | ✓ | ✗ | ✗ |
| **Missing variant handling** | ✓ | ✓ | ✓ | ✗*^a^* |
| **Multiallelic site handling** | ✓ | ✓ | ✗ | ✗ |
| **Automated visualizations** | ✓ | ✓ | ✗ | ✗ |
| **Phenotype analysis** | ✓ | ✗ | ✗ | ✗ |
| **Ancestry analysis** | ✗ | ✓ | ✗ | ✗ |
| **Layman-targeted documentation** | ✓ | ✓ | ✗ | ✗ |
| *^a^*Feature can be implemented by adapting other software functionality but is not explicitly documented as part of the PGM application workflow and will require expert understanding of the tool library. | | | | |

## Supplementary Table 2. Equivalent PGM application workflows among benchmarked tools.

Commands and parameters used to produce equivalent PGS values from benchmarked tools and the labeling of equivalent outputs.

| **Tool** | **PGM application command** | **Name of equivalent score in output data** |
| --- | --- | --- |
| **ApplyPolygenicScore** | apply.polygenic.score( missing.genotype.method = ‘normalize’) | PGS.with.normalized.missing |
| **PLINK2** | plink2 --score | SCORE1_AVG |
| **pgsc_calc** | nextflow run pgscatalog/pgscalc | AVG |
| **bigsnpr** | snp_match() snp_PRS() manual normalization | NA |

# Supplementary Figures

## Supplementary Figure 1. ApplyPolygenicScore benchmarking

**
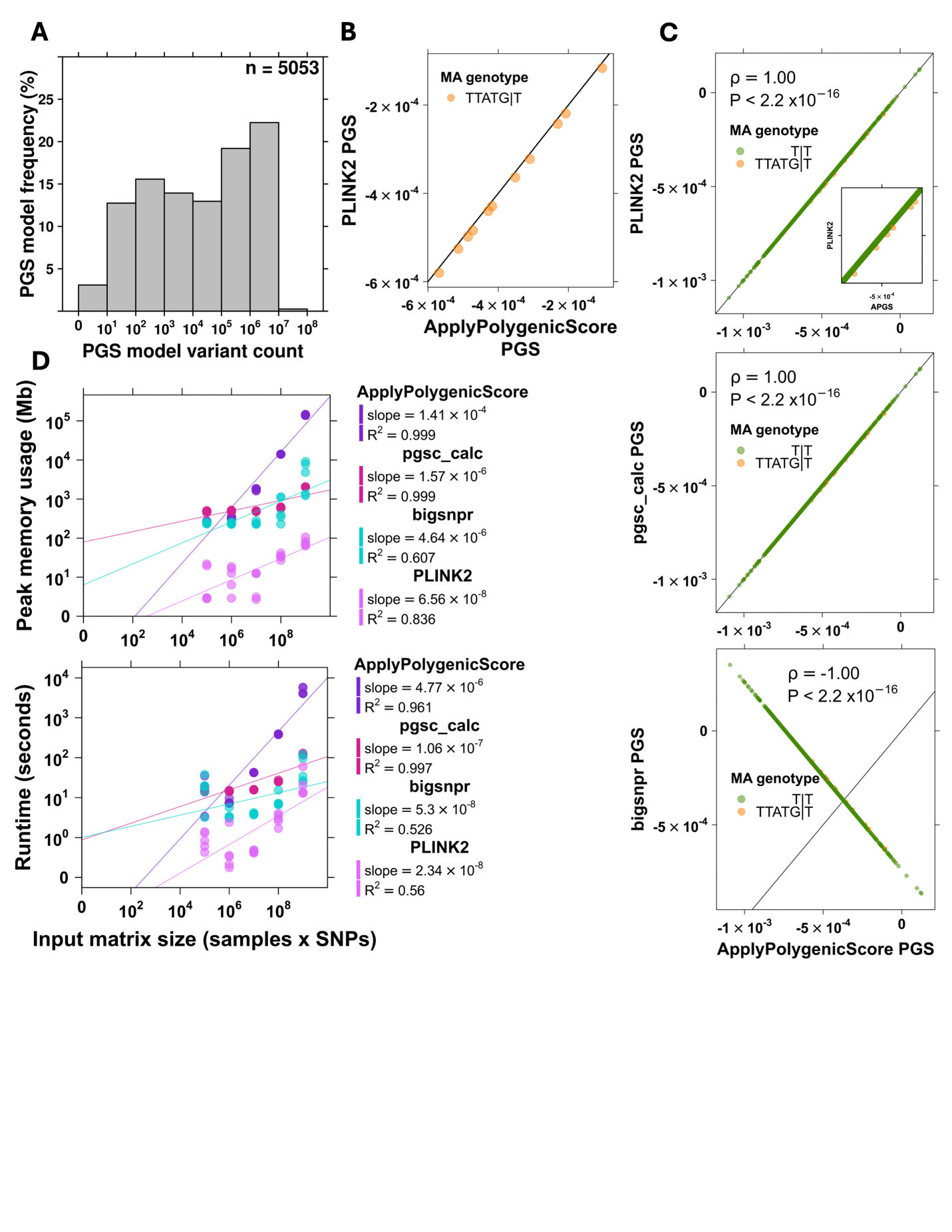
**

ApplyPolygenicScore v4.0.0 was benchmarked against three additional tools: pgsc_calc, PLINK, and bigsnpr. **A)** Distribution of polygenic risk score (PGS) model component variant counts in the PGS Catalog (release February 4, 2024). **B)** PGSs of individuals with a second alternative allele at a multiallelic site (MA) computed by PLINK2 and ApplyPolygenicScore from identical models. Black line indicates y=x identity. **C)** Correlation of computed PGSs from the same model and in the same dataset between ApplyPolygenicScore (APGS) and benchmarked tools. Black line indicates y=x identity. Point colors indicate genotype at a multiallelic (MA) site. **D)** Random access memory and runtime measured during equivalent polygenic risk model application workflows in benchmarked tools across increasing input size. Input size is defined as the number of samples multiplied by the number of variants in the applied model. Memory is defined as peak resident set size (RSS) measured by Nextflow tracing. Runtime is defined as realtime measured by Nextflow tracing. Lines and corresponding legends represent simple linear models fit to input size and reported statistic data for each tool.

## Supplementary Figure 2. Real data example of multiallelic site handling by ApplyPolygenicScore and PLINK.

**
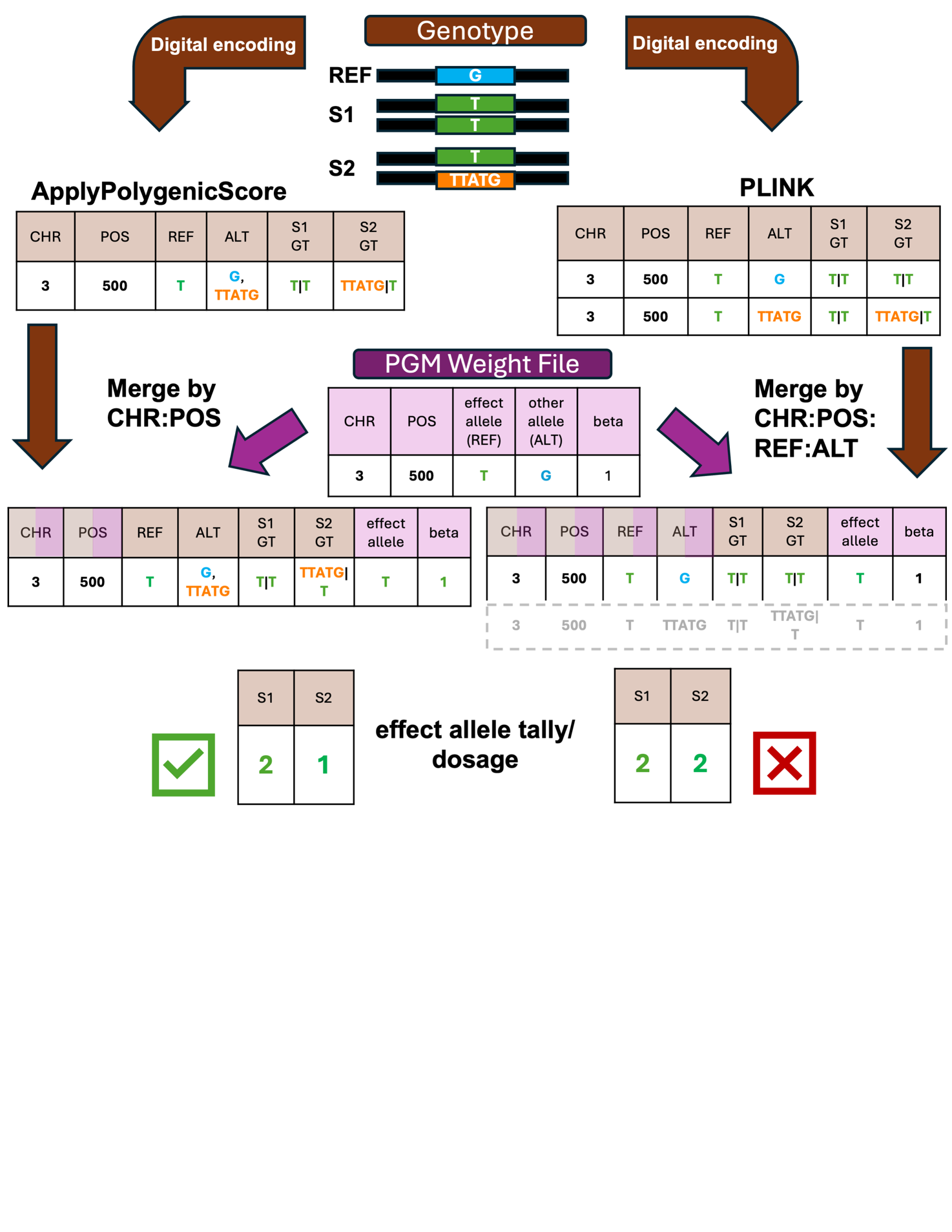
**

This diagram shows how effect allele dosage was computed at a multiallelic site. POS values have been shortened for brevity. A multiallelic (MA) site is a single genomic locus where more than two alleles are present across individuals in a cohort. *ApplyPolygenicScore* (APGS) and PLINK differ in multiallelic site handling, including in how site information is stored and how genotype and polygenic score model (PGM) weight data are merged. APGS strictly enforces the encoding of all alleles at a single coordinate in one line of a VCF, mimicking biology. PLINK encodes multiple alternate alleles at the same coordinate as multiple lines, creating duplicate sets of coordinates and forcing the inference of inaccurate allele combinations. A PGM weight file encodes weights that are allele specific. APGS matches PGM variants to genotypes using CHR and POS, maintaining one entry per genomic coordinate. PLINK matches PGM variants by CHR, POS, ALT and REF to avoid duplicate keys. This strategy is error-prone because there is no guarantee of concordance between the effect allele and either REF or ALT allele for all PGM variants. In the depicted PLINK case, one of the split entries for the MA site is never matched to a beta. APGS correctly counts effect allele dosage for both samples. For sample 2, PLINK incorrectly infers a genotype of T|T and counts a dosage of 2. S1 = Sample 1; S2 = Sample 2; CHR = chromosome; POS = genomic coordinate position; REF = reference allele; ALT = alternative allele; GT = genotype; PGM = polygenic score model.

## Supplementary Figure 3. BMI PGS and TCGA clinical features

**
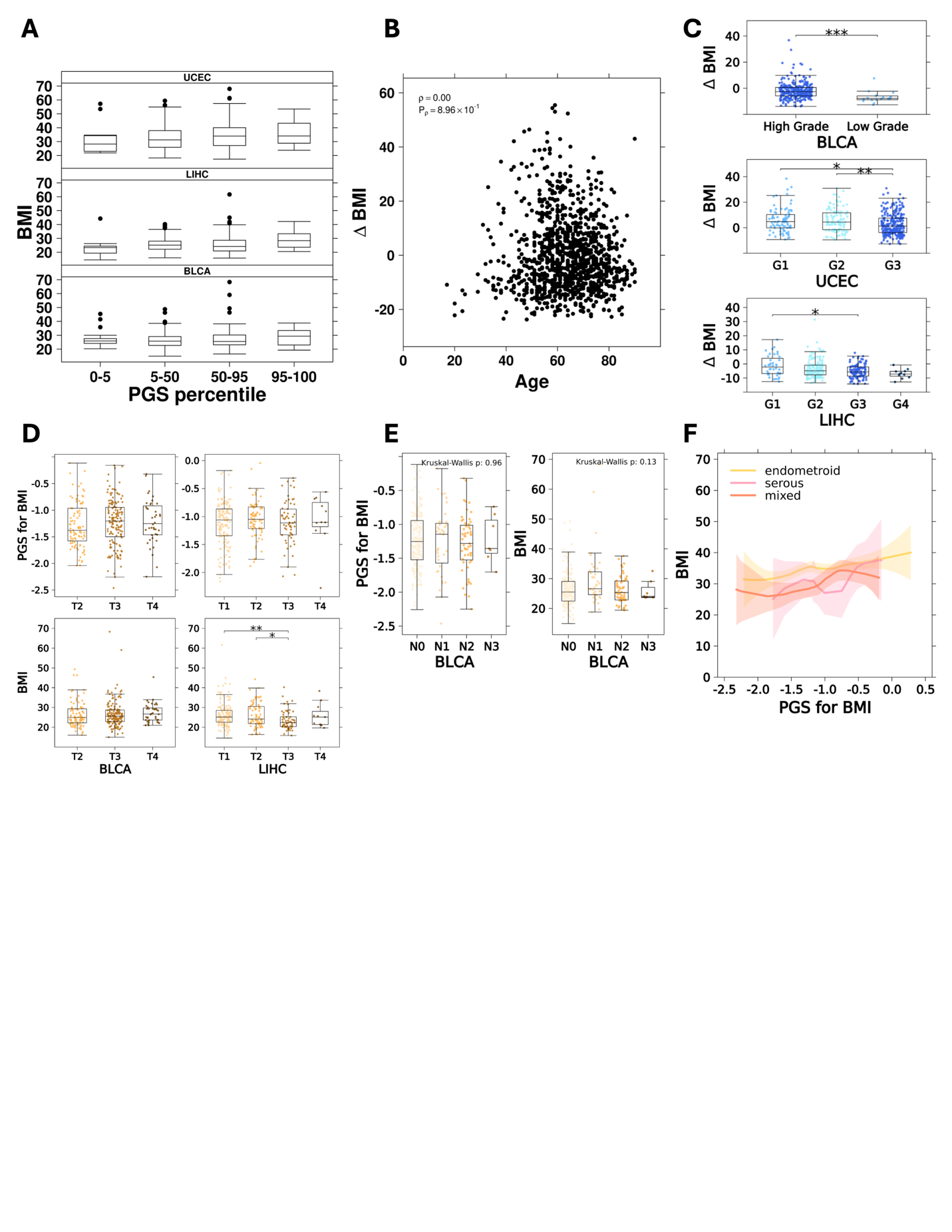
**

Analysis of BMI PGS computed by *ApplyPolygenicScore* in a case study of 1,071 individuals from the TCGA database, diagnosed with bladder (BLCA), liver (LIHC), or uterine (UCEC) cancer. **A)** Recorded BMI by percentile of PGS for BMI, separated by cancer type. **B)** Correlation between age at diagnosis and BMI delta, computed as residuals from a simple linear model with BMI PGS as a predictor of BMI. **C)** BMI delta by cancer grade**. D)** PGS for BMI and recorded BMI by T-category in bladder and liver cancer. **E)** PGS for BMI and recorded BMI by bladder cancer N-category. **F)** Loess-smoothed curves between PGS for BMI and PGS in men with UCEC, by histological subtype. In all boxplot panels depicted with asterisks, asterisks indicate statistically significant differences between indicated groups from pairwise Wilcoxon testing. P-values are adjusted for multiple testing within each set of groups using the Bonferroni method. One, two, and three asterisks indicate adjusted p-value below 0.05, 0.01 and 0.001 respectively.
